# Electronic Health Record Workflows in Acute Care Surgery: Ethnographic Study

**DOI:** 10.2196/89846

**Published:** 2026-09-28

**Authors:** Alex H Lee, Devesh Narayanan, Kristan Staudenmayer, Aussama K Nassar, Syed Morad Hameed

**Affiliations:** 1 Division of General Surgery, Department of Surgery, Stanford University, Stanford, CA, United States; 2 Division of General Surgery, Department of Surgery, University of British Columbia, 2775 Laurel Street, Vancouver, BC, V5Z 1M9, Canada, 1 604 675 3781; 3 Department of Management Science and Engineering, Stanford University, Stanford, CA, United States

**Keywords:** acute care surgery, ACS, electronic health records, EHR, decision-making, ethnography, risk assessment

## Abstract

**Background:**

Systematic strategies to harness electronic health record (EHR) workflows, reduce redundancy, and support decision-making remain limited in acute care surgery (ACS). Understanding how EHR systems and workflows intersect with time-sensitive settings is critical to improving decision-making and outcomes in ACS.

**Objective:**

This study aimed to evaluate how ACS clinicians leverage the EHR for decision-making and to identify opportunities and challenges for EHR-enabled decision support.

**Methods:**

We conducted a qualitative ethnographic study over a 6-month period, combining in-depth interviews with 15 ACS surgeons and providers and 100 hours of “paired fieldwork” observations spanning the entire perioperative arc by a surgical provider and an organizational sociologist. Using constructivist grounded theory, we identified the enablers, challenges, and opportunities for EHR-enabled decision-making in ACS.

**Results:**

Surgeons fell into two groups: (1) those who accepted information overload as inherent to the EHR, relying on generic templates and standard attestations, and (2) others who viewed it as a problem to fix, actively correcting errors and composing individualized summaries. Ambiguity in billing requirements drove overdocumentation, resulting in “note bloat” that obscured high-yield information. EHR use during decision-making focused primarily on risk assessment, though navigation challenges hindered access to critical data. Forecasting key outcomes that alter management or facilitate shared decision-making was seen as valuable. Automated risk stratification, generated from live EHR data while minimizing alert fatigue, was seen as a potential solution.

**Conclusions:**

ACS clinicians use various tactics to navigate EHR challenges and focus on high-value tasks. Streamlined risk assessment using EHR data may strengthen decision-making in critical moments, but solutions must integrate seamlessly within existing workflows to provide rapid and accurate outputs that prioritize meaningful outcomes.

## Introduction

Acute care surgery (ACS), which encompasses trauma, emergency general surgery, and surgical critical care, is thematically defined by the notion of surgical rescue, in which ACS teams must maintain a high state of anticipation for unexpected and potentially devastating events [1]. While ACS surgeons find purpose in the care and rescue of critically ill patients, they continue to face burnout at alarming rates as high as 67% [2,3]. A major contributor to this crisis is the escalating burden of administrative tasks, particularly the documentation of complex clinical data. Compared to their peers, ACS surgeons spend 60% more time on electronic health records (EHRs) [4-6]. The resulting cognitive overload undermines high-stakes decision-making while eroding their sense of purpose, ultimately impacting outcomes in rapidly evolving surgical emergencies [7].

Since the introduction of the Health Information Technology for Economic and Clinical Health (HITECH) Act [8] in 2009, EHRs have become the core infrastructure for data-driven technologies in health care. Yet EHR adoption continues to face challenges, including poor data standardization, interoperability issues, and difficulties integrating data into clinical workflows. While EHRs are widely used for documentation and billing, their role in supporting effective decision-making, particularly in acute settings, remains poorly defined. Moreover, few studies have evaluated the effectiveness of EHR-based tools in comparison to the bureaucratic counterfactual: the existing, nontechnical workflows that shape clinical practice [9,10]. Understanding the clinical and organizational context into which these digital tools are introduced is critical to ensuring they enhance, rather than disrupt, patient care, particularly in busy, high-stakes environments like ACS.

Accordingly, a broader sociotechnical perspective situates EHRs within complex clinical workflows, team interactions, and organizational constraints that shape their real-world use and impact [11,12]. Ethnographic studies have shown that clinical work is inherently distributed, iterative, and context-dependent, with EHR use reflecting adaptation to workflow complexity rather than deviation from intended design [13]. From this view, EHRs function as evolving information infrastructures, shaped over time by policy, institutional priorities, and user adaptation [14]. Ethnographic insights from field observations and stakeholder perspectives may therefore clarify how digital tools are embedded in practice, informing efforts to better support decision-making and surgical rescue.

Given that clinical decisions in ACS often occur in rapid, high-stakes settings without the benefit of preoperative planning, ACS represents a key opportunity to understand how EHRs and related workflows can be optimized [15]. This qualitative, ethnographic study aims to examine how ACS teams interact with EHR systems, focusing on how data are accessed, interpreted, and used in decision-making. By mapping existing workflows and patterns of engagement, we seek to identify barriers and opportunities for future implementation of digital tools that align with the realities of frontline surgical care.

## Methods

### Ethical Considerations

This study received ethical approval from the institution’s research ethics board (IRB-76999), and all participants provided informed consent. The study adhered to the SRQR (Standards for Reporting Qualitative Research) guidelines (Checklist 1) [16].

### Setting and Participants

This ethnographic study was conducted over a 6-month period between September 2024 and February 2025 at an academic teaching hospital providing level I trauma services. The institution’s ACS section accounts for approximately 11% of the hospital census and serves as a regional hub, coordinating care across 2 primary counties in collaboration with surrounding centers. The hospital uses Epic Hyperspace (Epic, Verona, and WI) as its front-end EHR interface, through which users document care, enter orders, review longitudinal patient data, and support billing workflows, all of which shape how they interact with patient data. Epic Systems has served as the institution’s primary EHR since its full implementation in 2008 as part of broader institutional efforts to modernize clinical infrastructure, improve care coordination, and support data-driven quality improvement and regulatory compliance initiatives.

Participants were recruited from the institution’s ACS section using a purposeful sampling strategy to capture variation across roles, experience levels, and EHR workflows. Attending surgeons (n=11) who were actively in clinical service during the study period were exhaustively sampled, with all eligible ACS faculty invited and included. Advanced practice providers (APPs; n=3) and one senior resident (n=1), identified during previous attending interviews as exemplary EHR users, were also purposively sampled to provide deeper insight into high-performing documentation practices and workflows within the section. In total, 15 ACS surgeons and providers participated in both semistructured interviews and direct observation of their EHR workflows (Table 1). The broader clinical context was captured through observational inclusion of approximately 20 other rotating residents and 5 fellows, who were not formally interviewed but participated in in situ ethnographic interviews during field observation. These brief targeted interviews were used to clarify observed behaviors and validate interpretations, consistent with established ethnographic methodology [17].

**Table 1.** Interview participant characteristics.

Participant group	Value, n	Sex	Years in practice
Attending surgeons	11	6 male, 5 female	0‐5 years: n=4; 6‐10 years: n=2; 11‐20 years: n=3; >20 years: n=2
APPs^a^	3	3 female	6‐10 years: n=2; 11‐20 years: n=1
Senior resident	1	1 male	—^b^

a APP: advanced practice provider.

b Not applicable.

### Data Collection

With growing calls from qualitative methodologists to conduct ethnographic fieldwork in interdisciplinary teams [18-20], this study developed and used a novel “pair fieldwork” technique to deepen and enrich our data collection. Specifically, a surgical care provider and an organizational sociologist conducted ethnographic in-depth interviews and field observations together throughout the project, to contrast insider (“emic”) and outsider (“etic”) perspectives on EHR practices. Each fieldworker focused on asking interview questions and writing ethnographic fieldnotes related to their areas of expertise; the surgical care provider focused on the nuances of clinical decision-making, whereas the organizational sociologist focused on routines, workflows, and broader organizational processes. As a result of these differing emphases, the 2 fieldworkers’ notes diverged significantly even while observing the same phenomenon: the surgeon’s notes tended to contain details on how patient data were accessed, discussed, and integrated into real-time clinical decision-making, whereas the sociologist’s notes tended to contain details on broader sociotechnical factors (eg, pace of work, division of labor, and the broader spatiotemporal context of clinical encounters) that shaped EHR use. To maximize the analytic value of this variation, the 2 fieldworkers maintained independent fieldnotes and descriptive memos throughout the study, while meeting regularly with the research team to discuss high-level similarities and differences in what was being observed, and to strategize approaches to broaden and deepen data collection. Moreover, since neither fieldworker held clinical or supervisory roles within the section or department, both could move freely in the field and prompt open, candid discussions of EHR practices without appearing to be implicated in the section’s organizational hierarchy or reporting structures.

An interview protocol was developed and iterated by the research team a priori, beginning with general questions about participants’ daily workflows, and subsequently covering questions about their practices, preferences, and needs related to generating, inputting, analyzing, and interpreting EHR data, as well as general perceptions of EHR workflows and existing systems. Participants were recruited through an internal faculty and staff email list as well as announcements during section meetings. Interviews were conducted either in-person or on a video conference platform (Zoom, version 6.2) depending on participant availability and were voice-recorded with consent for subsequent transcription.

Approximately 100 hours of field observations were conducted during the study period. In keeping with best practices in constructivist (as opposed to purely inductive) grounded theory [21-23], observation scripts were developed to tactically focus fieldwork toward understanding the processes and workflows underlying this study’s phenomenon of interest: how ACS clinicians interact with EHR data. First, sites were identified where ACS teams regularly interacted with EHR data when making clinical decisions—most notably, daily handover conferences, patient rounds, clinics, and consultations. The 2 fieldworkers regularly visited these sites over a 6-month period, documenting interactions, actions, and behaviors, as well as broader contextual information surrounding interactions with the EHR. As data collection progressed, observation scripts were refined to also include sites where individual clinicians input and interpret EHR data—attendings’ private offices and the ACS resident or APP “team rooms.” The diversity of environments, spanning intrasettings and multidisciplinary settings, enabled the capture of workflows and interactions across both hierarchical and nonhierarchical contexts. At these sites, a “think-aloud” protocol was used, whereby individuals were asked to vocalize what they were thinking as they interacted with the EHR, to capture their beliefs, preferences, and attitudes on the role of the EHR in clinical decision-making [24,25]. Throughout our fieldwork, passive observation was interspersed with ethnographic interviews to confirm the accuracy and veracity of our observations, as well as to reflect on specific anomalies and inconsistencies that arose during the observations.

### Data Analysis

Data analysis was iterative and proceeded along 3 broad stages [26,27]. The first stage entailed open coding, where the research team stayed close to the textual content of fieldnotes and interview transcripts to identify patterns related to how clinicians recorded, interpreted, and leveraged EHR data. At this stage, multiple phenomena of interest were identified as occurring frequently and/or being putatively important to the study participants. Subsequently, fieldnotes and interview transcripts were reanalyzed with a focus on generating explanations and underlying mechanisms for each phenomenon identified in the previous stage. Finally, in the third stage of theoretical coding, codes that had reached thematic saturation were abstracted and connected with extant literature to identify novel contributions to research on EHR workflows and their impact on clinical decision-making [28]. All analyses were performed using ATLAS.ti (version 25.0; Lumivero).

## Results

### EHR Use During Clinical Shifts

On average, participants reported spending approximately 3 to 4 hours per 12-hour shift engaged with the EHR, both by reviewing notes on the fly during patient rounds and by more deliberately reviewing and discussing patient information during structured settings such as handover conferences. However, considerable heterogeneity was observed in terms of how this time was spent, reflecting underlying differences in perspectives on how to input, retrieve, and leverage EHR data for clinical decision-making.

### Perspectives on Inputting Data Into EHR Systems

Two distinct types of EHR users, in terms of their approach to inputting data into EHR systems, were identified among clinicians (Textbox 1). One group, labeled the “passive acceptors,” perceived the EHR as a burdensome task to be efficiently circumvented for clinical work. They tended to use generic templates for documentation and standard attestations, such as writing “as above,” while accepting minor errors in notes as is. Passive acceptors were also less likely to provide feedback regarding notes (eg, to trainees). On the other hand, “active editors” viewed the EHR as a systems problem to fix and leverage for clinical work. They often generated individualized summaries and attestations, while identifying and correcting minor errors in the EHR for accurate communication.

Textbox 1. **Illustrative quotes describing key characteristics of electronic health record (EHR) users in acute care surgery (ACS).**

**Passive acceptors**

- EHR is a barrier to circumvent for clinical work
  - **Nurse Practitioner:** “I know that nobody is going to read my note fully, so it really matters more how I present my information during rounds.” (Field observation: handover conference)
- Generic templates and standard attestations are preferred
  - **Attending:** “I always click ‘moderate [severity]’ for most floor patients. [‘Moderate’ is one of the options for severity required for billing] I always put ‘moderate’. Dr XX is really the expert and will show you how to really charge things. I just put ‘moderate’ all the time.” (Field observation: clinic)
- Minor errors are left as is for efficiency
  - “When I close wounds, I’m very particular about how they’re closed. I don’t use staples on skin … I saw a note … that said ‘closed with staples.’ I knew for a fact that the skin was not closed with staples, but it was carried over from some other formatted note. But I didn’t have time to correct it nor did I tell [the author].” (In-depth interview: attending)
  - “I would say there are some attendings that require more detail. They actually will read the note and then text me about it… they’ll give you a little bit of feedback. But for the most part, most attendings don’t end up giving you any.” (In-depth interview: resident)

**Active editors**

- EHR is a problem to solve and improve on
  - “I enjoy going on the EHR when I'm reading about patients, making quick decisions, running surgery… That works well in those situations—in terms of accessing notes, labs, x-rays, consultant notes.*”* (In-depth interview: attending)
- Customized, individualized summaries are preferred
  - **Attending:** “Look at the whitespace, the clear headings, and the summary at the end. I don’t mean to be biased, but I think this is quite neat, no? I’m quite proud of it … I made a template when I joined, and I’ve been trying to improve it ever since.*”* (Field observation: clinic)
- Errors are identified and fixed
  - [Attending calls out an error on a note, recognizing an erroneous diagnosis that was recorded] “Make sure this goes into the note, please.” (Field observation: patient rounding)
  - “There are things that get missed because things get carried on from prior days and they don’t read them. So, I have to go over them … read and make sure there’s pertinent information that is relevant to patient care … You go over and make sure it’s accurate, make sure it’s up to date.*”* (In-depth interview: attending)

Importantly, these 2 categories (“passive acceptors” and “active editors”) imply general tendencies, rather than rigid, determinate labels—and, as such, were responsive to broader sociotechnical factors surrounding EHR use (Figure 1). For instance, during particularly busy shifts or when dealing with routine, repetitive cases, surgeons who may otherwise tend to be active editors may, in the interest of time, adopt practices similar to passive acceptors. Similarly, when dealing with particularly complex cases or when reviewing notes written by inexperienced interns or students, ordinarily passive acceptors may adopt practices similar to active editors. Tenure appeared to be another important factor in shaping these tendencies. Senior attendings with more experience writing and reviewing notes tended to have detailed templates and routinized practices to cover a wider range of cases (thus tending toward passive acceptance), whereas junior attendings tended to spend more time customizing their summaries and fussing about minor errors (thus tending toward active editing).

Next, when surgeons were explicitly prompted to reflect on sources of documentation errors and overdocumentation, or “note bloat,” opaque billing requirements were identified as a key driver of inaccuracies, cognitive stress, and overall challenges with using the EHR across both groups (Textbox 2, theme 2.1). Strategies to overcome these documentation burdens, such as copying and pasting text from the previous day, further contributed to the vulnerability of information to errors (Textbox 2, theme 2.2).

**Figure 1.**
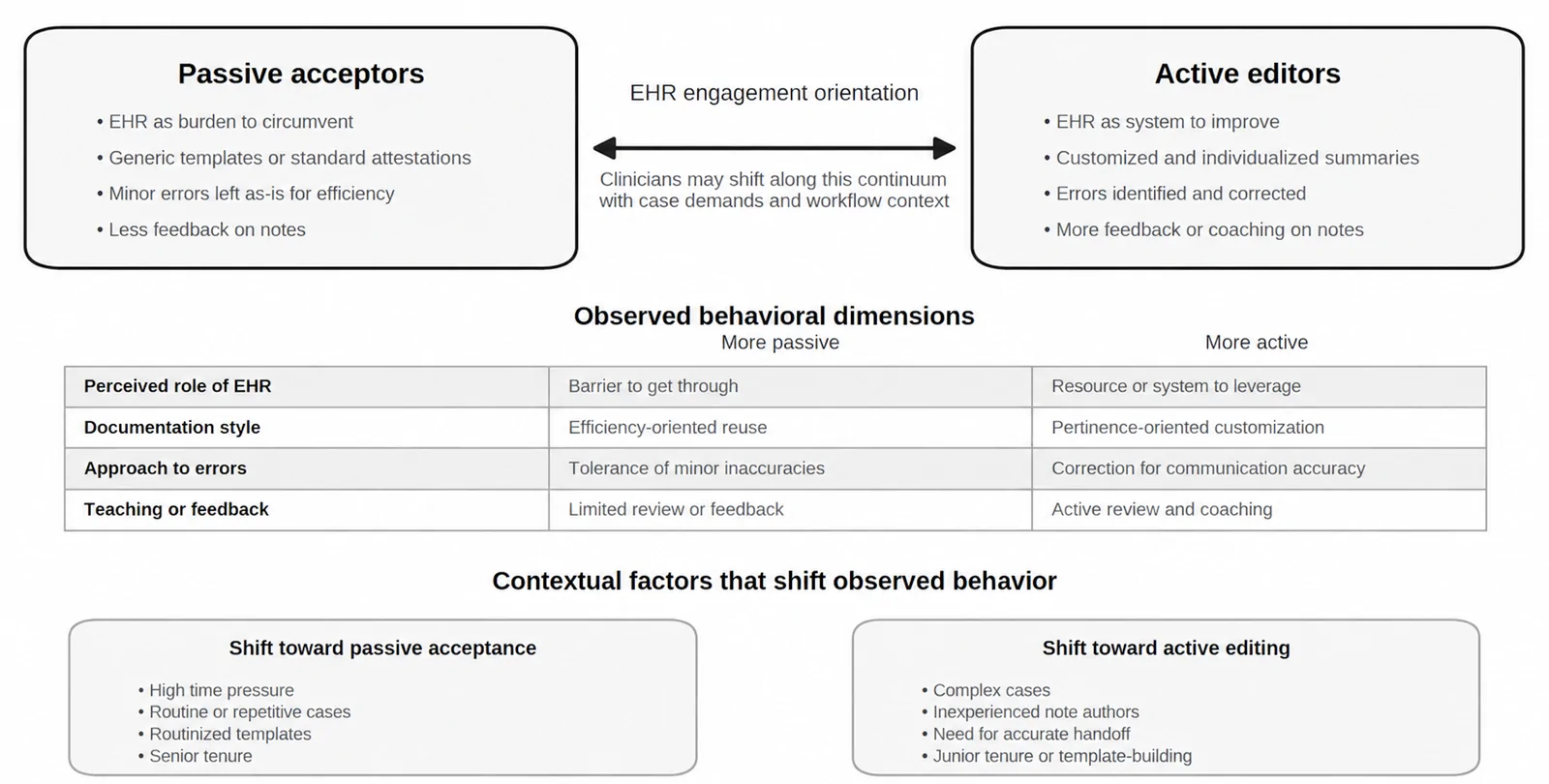
Continuum of clinician engagement with electronic health record (EHR) documentation in acute care surgery (ACS). Passive acceptors and active editors represent tendencies rather than fixed user types. Clinicians could shift along the continuum depending on contextual factors such as time pressure, case complexity, author experience, handoff needs, and the degree of template routinization.

Textbox 2. **Illustrative quotes describing sources of overdocumentation and errors.**

**Theme 2.1: Opaque billing requirements are a major driver of overdocumentation and note bloat**

- “What is a good note? A good note is one that meets billing requirements; no more, no less!” (In-depth interview: attending)
- “When I made the standardized templates for progress notes, I tried to go through all the [financial-legal] requirements and hit them all. That’s why the templates are made that way. Like the review: ‘you need to have a certain number of physical exam systems’…. It’s very odd because none of it is very clinical; they’re very arbitrary rules! For instance, you need to add a skin exam as part of a distinct system, otherwise it doesn’t count. How does that make sense?” (In-depth interview: attending)
- “For operative notes, now they’re making us add all this stuff that I really don’t think is necessary. If you do a debridement, you have to say how much you debrided it, how deep did you debride it. I’m pretty sure those aren’t requirements, but our coders are convinced that they are. So, we’re stuck…. Then you get peppered with additional messages in your inbox.” (In-depth interview: attending)

**Theme 2.2: Attempts to mitigate documentation burdens (eg, copyforwarding) contribute to documentation errors**

- **Observer:** “What happened there? Wasn’t it in the note [that the patient’s feeding tube was already removed]?” **Resident:** “It was! [Scrolls down the note] I put it here in the assessment and plan, but I think I copied over yesterday’s note that still showed the tube was in. It is honestly quite tedious to check and edit that every time.” (Field observation: ACS team room)

- “After copying a note, I’ll edit the note a little bit, but it’s so burdensome…. Having to sift through pages of notes to edit things takes forever, and so it’s not worth the time. And so, you'’ll find errors [in copy-forwarded notes] compound upon each other. If people tried to edit every note, they would spend exponentially more time on the computer than with patients.” (In-depth interview: attending)

### Perspectives on Retrieving and Interpreting Data From EHR Systems

Participants noted that, unlike other surgical specialties where surgeons often have the time to review EHR data in detail and plan operative strategies in advance, ACS demanded faster, more reflexive approaches to retrieving and interpreting useful information. Accordingly, a primary motivation for leveraging EHR data in ACS was to support real-time decision-making, often through streamlined risk assessment (Textbox 3, theme 3.1). For instance, observations revealed that attending surgeons frequently raised questions or comments regarding the patient’s status and trajectory to decide on operative intervention. During interviews, participants also emphasized the role of EHR data in supporting risk assessment.

Textbox 3. **Illustrative quotes describing EHR use for decision-making in ACS.**

**Theme 3.1: Data from the EHR is frequently leveraged and important for risk assessment**

- [The attending mentions using the Emergency Surgery Score to risk stratify each consult. The residents seem to be responsible for these tasks at this time, but the attending states that they are trying to “streamline it using the EHR”] (Field observation: handover conference)
- [Many questions, especially from the attendings, are around patient status and trajectory. “How much pressors are they on,” *”*How are their labs looking like now,” “Can we book them for surgery today.” These questions lead to decisions around surgery…. Sometimes, the case presenter is wrong on specific laboratory values or medication doses. The presenter tries to find the specific information on the EHR but usually has trouble finding it right away.] (Field observation: handover conference)
- **Attending:** “When we do morning rounds and go through a large number of cases and charts, is there a way we can quickly stratify risk?” [Both residents and attendings respond positively. One attending brings up how even when dealing with similar conditions, [depending on specific patient characteristics], one patient would have “a longer window for rescue” (eg, a young, previously healthy patient) compared to an older or frailer patient.] (Field observation: handover conference)

**Theme 3.2: Note bloat limits extraction of high-value information for decision-making**

- “In the jumble that is [the EHR], what happens is you have a little bit of narrative at the beginning, then you have some physical findings. Sometimes, you'’ll have long streams of laboratory data, investigations, and medications, relevant or not. You can scroll all the way to the bottom and realize it just trails off and there’s no summary at the end. Then you scroll back up to find somewhere in there will be the clinical summary for that day.” (In-depth interview: attending)

- **Attending:** “Let me find the ICU note…. It’s a bit hard to tell which one the ICU note is. Think it’s this one.” [Clicks on one of the notes] “I don’t love the way it looks.” [He begins scrolling down, and there is a long list of tests, exams, and summaries of events] “I don’t know if human beings are meant to understand data like this! There’s no prioritization here, and I don’t know what to focus on.” [He scrolls to the bottom of the note] “And then it just ends all of a sudden! There’s no impression or plan!” (Field observation: patient rounding)

- [The attending asks about the patient’s medical history to the resident on the EHR] **Resident:** “Hmm, where is it?” [Scrolls down to the end of the note] “Not here, I think. Ugh, it is so embarrassing to have you all watch me do this!” [She opens up a new note and scrolls to the bottom]. “Not here either.” [Grunts in frustration]. **Attending:** “That’s fine. We’ll look for it in case it comes up during rounds later.” (Field observation: handover conference)
- [For the next patient, there was a discrepancy between what was documented and the working diagnosis described by the resident. She originally stated that this patient had a cecal perforation, but on review of the operative note by the attending, the patient actually had a small bowel perforation. The attending calmly corrected this. He asked the team to further review the patient’s chart and requested to keep track of the patient’s hemodynamics and laboratory trends, establishing clear criteria for when to escalate care.] (Field observation: patient rounding)

Note bloat and errors that propagated through copy-forwarded documentation posed significant challenges for ACS clinicians, making it difficult to identify pertinent information, especially given the rapid pace at which data needed to be reviewed and interpreted (Textbox 3, theme 3.2). While surgeons hoped that EHR-enabled decision support would leverage objective data and mitigate the reliance on maladaptive heuristics, the errors and note bloat pervading the EHR made it difficult to effectively integrate data into decision-making workflows.

Moreover, as risk assessment emerged as a key use of EHR data, several surgeons reported relying on existing risk-stratification systems, including standalone calculators. However, they also pointed to several shortcomings in these tools (Textbox 4, theme 4.1).

Textbox 4. **Illustrative quotes describing the use and shortcomings of existing decision support systems.**

**Theme 4.1: Existing tools are not optimized for electronic health record (EHR) integration**

- “I use this NSQIP risk calculator…. It’s kind of not ideal for the emergency surgery population. It accounts for a broad-based, more so elective practice. You could look up the literature, but it tends to misestimate emergency surgery patients and [those who] have multiple comorbidities and what not.” (In-depth interview: attending)
- “There are calculators out there that help you with decisions. None of them are perfect…. I’m always like, how come these tools aren’t embedded into [the EHR] so that I’m able to open patients’ charts and click on the [risk calculator] tab. It should be like this – with the calculator saying this, automatically.” (In-depth interview: attending)

### Perspectives on Future Opportunities for Improving EHR-Enabled Decision Support

When asked to describe the ideal EHR-enabled decision support tool during in-depth interviews, participants conceptualized an embedded risk assessment model that forecasted key ACS outcomes. They emphasized the need for automated data input to eliminate the time-consuming task of manually identifying and entering key information (Textbox 5, theme 5.1). Just as critically, they stressed that model outputs should not increase cognitive burden, for example, by contributing to alert fatigue, but instead should be easily accessible and intuitive (Textbox 5, theme 5.2). Importantly, clinicians underscored that any embedded tools must have clinical utility (Textbox 5, theme 5.3). For instance, the most valuable predictions were those that systematically heightened vigilance and better standardized escalations in care, as well as those that conveyed overall outcomes that would help guide shared decisions around operation.

Even as ACS surgeons expressed enthusiasm for incorporating specific tools into the EHR, they emphasized that successful development and implementation must align closely with existing workflows. Surgeons highlighted that risk assessment, for example, should be grounded in an understanding of which outcomes may be most useful to predict, the extent to which different risk thresholds may or may not warrant revised management plans, and how these tools can be leveraged to discuss treatment options with patients.

Textbox 5. **Illustrative quotes describing electronic health record (EHR)-enabled decision support.**

**Theme 5.1: EHR-enabled risk assessment should be automated using real-time data**

- “I can manually enter data. But it’s not useful. It’s because of the pace of work, it’s not feasible to calculate scores…. For example, for liver failure, there is a score called MELD score which will tell you a lot about that patient’s risk. The problem is the MELD score is a bit difficult to calculate.” (In-depth interview: attending)

- “I had to pull my phone, do the calculation and then decide. But [the EHR] should be able to make a suggestion to me, right? It has the data that I’m using. It should be doing it automatically. In fact, I feel like it should automatically suggest it and just be like, this is what we decided, do you agree or disagree? All these things can be automated. Why am I doing this manually in 2024?” (In-depth interview: attending)

**Theme 5.2: Models should be embedded into the EHR as a dashboard without exacerbating alert fatigue**

- “If the data could be displayed in a user-friendly way…. People carry paper notes and lists right now, but [the EHR] is how I see patients and they have these little icons, but I actually don’t know what those icons are. If there is a way that I could absorb all the pertinent information quickly, that would be valuable.” (In-depth interview: attending)
- “All of these indices, there’s that constant tension between being overly sensitive. Sometimes it over triggers. And then you run the risk of alarm fatigue, because people see this, and they just kind of become immune to it.” (In-depth interview: attending)

**Theme 5.3: Models should be tailored to output relevant and/or actionable outcomes in ACS**

- “I think the stakes are high in these patient populations, so mortality immediately comes to mind, but I think organ failure is another important outcome…. I care about their hospital length of stay—it’s a marker for how well you care for them. And then, of course, functional outcomes are ultimately the most important thing, but we don’t really have much information about that.” (In-depth interview: attending)
- “Could we get all of the data from the EHR, use AI to see if we could build a predictive tool based on the information available to predict which patients were going to vomit, which patients were going to breathe it into their lungs, and which ones would wind up in the ICU? That’s something that’d be helpful, because you’re being very specific, and your [risks] are then actionable.” (In-depth interview: attending)
- “Things like discharge to a nursing facility. I think a lot about those longer-term complications that affect people’s lives…. People care if they’re going to be the same mentally… or what their life is going to look like in 6 months, or something like that. That’s what people care about.” (In-depth interview: attending)

## Discussion

This ethnographic study examined the EHR workflows through which ACS clinicians make data-driven decisions, identifying both opportunities and barriers to implementing digital tools that support surgical care delivery. Opaque billing requirements were a key driver of documentation overload, contributing to note bloat and errors. ACS clinicians responded to this burden either passively or actively. Decision support through risk assessment emerged as a central use of EHR data, with clinical decisions hinging on the timely identification and integration of relevant data points that could facilitate surgical rescue. Clinicians emphasized that digital tools must be designed with attention to data input, output, accuracy, and clinical relevance to augment, rather than impede, effective decision-making. These findings align with broader sociotechnical perspectives on health informatics, showing that system performance emerges from interactions among technical design and the social, organizational, and cognitive contexts of use.

ACS may be viewed as an extreme environment in which high acuity, uncertainty, and time pressure, compounded by worsening workforce shortages, magnify the impact of documentation burden and note bloat [4-6,29]. Yet, prior work suggests that such extreme contexts can serve as revealing cases, exposing latent inefficiencies that might otherwise remain obscured, as constraints of time, uncertainty, and limited resources amplify their effects [30]. In this study, these dynamics were evident in users selectively bypassing bloated notes, relying on synchronous communication, or developing workarounds to rapidly locate high-value information. These behaviors highlight how excessive or poorly structured documentation can hinder information retrieval and clinical decision-making. Although such adaptations may be particularly necessary in ACS, they likely reflect broader, systemic challenges in EHR use, where the consequences of documentation burden may be less immediate but remain consequential.

The competing demands of administrative compliance and clinical care have shaped the evolution of EHRs from single-purpose clinical tools into multipurpose systems. The HITECH Act, for example, catalyzed EHR adoption by strengthening the financial rationale for implementation among hospital administrators [31]. Since then, documentation standards designed to support financial and legal compliance, such as those related to Current Procedural Terminology (CPT) codes and the *International Classification of Diseases* (ICD), have increasingly influenced digital and clinical workflows alongside persistent interoperability and data fragmentation challenges [32]. Such requirements have further exacerbated misalignment between system-imposed documentation structures and the cognitive and workflow needs of frontline clinicians [12,14].

This is particularly consequential for medically complex patients, where, as Naik and Singh [33] emphasize, asynchronous communication methods such as the EHR are critical for transmitting pertinent information across multiple receivers without interruption. In ACS, there is a natural bias toward synchronous, interruptive communication (eg, face-to-face conversations and phone calls) due to the time-sensitive nature of care. While necessary and efficient in urgent or life-threatening situations, overreliance on synchronous communication alone can lead to cognitive overload, increased dependence on short-term memory, work-related stress, and higher risk of medical errors [34,35]. Ethnographic studies of EHR use have similarly shown that clinicians rely on both formal documentation and informal communication practices to manage complex, time-sensitive care, with breakdowns often occurring when digital systems fail to support this distributed coordination [13]. In ACS, there is thus a strong incentive to develop noninterruptive EHR systems aligned with surgeon workflows to support shared decision-making, with prior work demonstrating that targeted EHR-based redesign can improve timeliness of care [36].

While greater clinical experience and seniority might suggest a shift toward intuitive decision-making over time, previous research shows that surgical judgment, particularly in assessing risks and benefits, remains highly variable regardless of experience level, especially in areas lacking clear guidelines [37,38]. As a result, EHR-enabled decision support tools are increasingly valued for their ability to promote deliberative reflection and counteract maladaptive cognitive biases and heuristics [39]. Harnessing this potential will therefore require documentation practices that balance efficiency with the effective inclusion and retrieval of clinically important information.

A key strategy may be to shift from systems-based to problem-based charting. Interviews revealed that documentation standards for billing are often poorly understood, with some believing that systems-based documentation is required for certain services when it is not. Increasing transparency around billing requirements could help dispel these misconceptions and encourage more focused documentation of high-value information without compromising financial-legal standards. Accordingly, a previous study of problem-based charting demonstrated significant improvements in capturing certain critical care diagnoses for billing purposes [40]. Such a transition would facilitate the identification and use of relevant EHR information to support surgical and perioperative decision-making.

In this study, EHR data were often leveraged for risk assessment, such as to support critical decisions around when and whether to operate. However, the manual search and entry of pertinent information remain significant barriers to adopting EHR-embedded decision support tools, indicating “hidden work” in the form of cognitive and navigational tasks not accounted for in system design [41]. Recent advances in AI, including models capable of generating real-time predictions or recommendations, have made it increasingly feasible to overcome these limitations [42,43]. These insights must also be clinically meaningful or actionable, particularly in settings where decision-making already depends on heterogeneous or uncertain clinical data [44]. For example, predicting specific complications such as postoperative respiratory failure should be coupled with standardized, risk-stratified protocols that align predicted risk with appropriate interventions, such as tailored monitoring and recovery strategies. In contrast, predictions of more general outcomes like mortality or overall morbidity may be most useful in situations of clinical uncertainty, supporting decisions and shared decision-making during equipoise through quantitative data, or serving as a second opinion when clinicians seek additional validation. Analogous decision support frameworks in other high uncertainty domains have used fuzzy multicriteria decision-making models to integrate expert judgment, conflicting criteria, and ambiguous data, illustrating the broader relevance of structured approaches when decisions cannot be reduced to a single quantitative output [45,46].

While there are certain perceptions that clinical gestalt may ultimately override any outputs from EHR-enabled decision-making, there is also general agreement that digital workflows can be better optimized to mitigate human cognitive biases [47]. For example, studies have demonstrated improved predictive accuracy when surgeons are supported by risk calculators, and, in some cases, the tools alone can outperform both unaided and aided surgeons [48,49]. This underscores the importance of educating clinicians to meaningfully integrate decision-making processes into digital workflows without falling into patterns of overreliance or reflexive skepticism. One suggested model is a Bayesian-like approach, where AI output is treated as an informed second opinion from a colleague with a distinct expertise profile, neither overriding nor subservient to clinical judgment, but contributing to a well-calibrated decision [50]. Translating this model of collaboration into routine practice will require prospective validation across dynamic environments, subgroup populations, and ACS care models, with attention to calibration and clinically actionable outcome definitions. Clinician trust will also depend on whether outputs are interpretable, available at the right moment in the workflow, and linked to clear responsibilities and care pathways. To ensure these tools augment care delivery, future implementation should therefore evaluate not only predictive performance, but also effects on alert fatigue, documentation burden, communication patterns, and unintended changes in surgical decision-making.

This study has several limitations. First, as a single-center ethnographic study, findings may not be generalizable to institutions with different EHR systems, workflows, or models of ACS care. Documentation practices are also shaped by local institutional culture, remuneration and billing structures, and national regulatory context, which may further limit transferability. However, several findings likely reflect broader sociotechnical dynamics relevant beyond academic hospitals, including documentation burden, note bloat, reliance on workarounds, difficulty retrieving high-value information, and the need for decision support tools embedded within existing workflows. In nonacademic hospitals or smaller health systems, these challenges may be amplified by leaner staffing models, fewer APPs or trainees, and less information technology support, although simpler organizational structures may also facilitate more direct communication. Thus, while the specific EHR behaviors observed in this study should be interpreted in context, the broader need to align documentation, information retrieval, and decision support with frontline clinical workflows is likely applicable across diverse acute surgical care settings. Second, the presence of observers may have introduced reactivity or response bias, and any concurrent EHR-related initiatives may have influenced participant perspectives. Third, our purposive sampling strategy may have introduced selection bias and underrepresented certain groups, including those with limited availability (eg, night-shift staff) and early-career providers. In addition, we did not fully capture EHR interactions involving consulting services or bedside nursing staff. Nevertheless, the inclusion of all attending surgeons alongside APPs and trainees, combined with a dual-observer fieldwork approach and in situ ethnographic interviews, supports the breadth and validity of the findings. Finally, while we identified opportunities for EHR-enabled decision support, we did not evaluate the implementation or impact of such tools, which will require future prospective and intervention-based research.

EHR systems are critical parts of the ACS workflow, shaping how information is documented as well as how decisions are made. This study highlights the dual burden of administrative documentation and clinical complexity, demonstrating that EHR practices are both a source of friction and a potential site for innovation. Meaningful improvement will likely require reconsideration of how documentation, communication, and decision-making processes are structured within the broader clinical system. While ACS clinicians vary in their engagement with the EHR, there is broad recognition that better integration of clinically valuable decision support, tailored to the ACS context, could improve surgical workflows and patient outcomes. Aligning EHR design, documentation standards, and digital tools with frontline ACS workflows is essential to advance a more efficient, accurate, and cognitively supportive environment for high-stakes surgical care delivery.

## Supplementary material

10.2196/89846 Checklist 1 SRQR checklist.

## Abbreviations

ACS: acute care surgery
APP: advanced practice provider
CPT: Current Procedural Terminology
EHR: electronic health record
HITECH: Health Information Technology for Economic and Clinical Health
ICD: *International Classification of Diseases*
SRQR: Standards for Reporting Qualitative Research
