# Supplementary material for: Electronic Health Record Workflows in Acute Care Surgery: Ethnographic Study
Source: JMIR Med Inform. 2026 Sep 28;14:e89846. doi: 10.2196/89846 (PMC13618404; doi:10.2196/89846)
Supplement: Supplementary file 1 [file medinform-v14-e89846-s001.doc]

**Online Resource 1. Standards for Reporting Qualitative Research Checklist**

**Standards for Reporting Qualitative Research (SRQR)**

O’Brien B.C., Harris, I.B., Beckman, T.J., Reed, D.A., & Cook, D.A. (2014). Standards for reporting qualitative research: a synthesis of recommendations. *Academic Medicine, 89(9)*, 1245-1251.

| **No. Topic** | **Item** | **Page #** |
| --- | --- | --- |
| **Title and abstract** |  |  |
| S1 Title | Concise description of the nature and topic of the study identifying the study as qualitative or indicating the approach (e.g., ethnography, grounded theory) or data collection methods (e.g., interview, focus group) is recommended | 1 |
| S2 Abstract | Summary of key elements of the study using the abstract format of the intended publication; typically includes objective, methods, results, and conclusions | 2 |
| **Introduction** |  |  |
| S3 Problem formulation | Description and significance of the problem/phenomenon studied; review of relevant theory and empirical work; problem statement | 4-5 |
| S4 Purpose or research question | Purpose of the study and specific objectives or questions | 5 |
| **Methods** |  |  |
| S5 Qualitative approach and research paradigm | Qualitative approach (e.g., ethnography, grounded theory, case study, phenomenology, narrative research) and guiding theory if appropriate; identifying the research paradigm (e.g., positivist, constructivist/interpretivist) is also recommended | 5 |
| S6 Researcher characteristics and reflexivity | Researchers’ characteristics that may influence the research, including personal attributes, qualifications/experience, relationship with participants, assumptions, or presuppositions; potential or actual interaction between researchers’ characteristics and the research questions, approach, methods, results, or transferability | 5-6 |
| S7 Context | Setting/site and salient contextual factors; rationale^a^ | 5 |
| S8 Sampling strategy | How and why research participants, documents, or events were selected; criteria for deciding when no further sampling was necessary (e.g., sampling saturation); rationale^a^ | 5 |
| S9 Ethical issues pertaining to human subjects | Documentation of approval by an appropriate ethics review board and participant consent, or explanation for lack thereof; other confidentiality and data security issues | 5 |
| S10 Data collection methods | Types of data collected; details of data collection procedures including (as appropriate) start and stop dates of data collection and analysis, iterative process, triangulation of sources/methods, and modification of procedures in response to evolving study findings; rationale^a^ | 5-7 |
| S11 Data collection instruments and technologies | Description of instruments (e.g., interview guides, questionnaires) and devices (e.g., audio recorders) used for data collection; if/how the instrument(s) changed over the course of the study | 5-7 |
| S12 Units of study | Number and relevant characteristics of participants, documents, or events included in the study; level of participation (could be reported in results) | 5-7 |
| S13 Data processing | Methods for processing data prior to and during analysis, including transcription, data entry, data management and security, verification of data integrity, data coding, and anonymization/deidentification of excerpts | 7 |
| S14 Data analysis | Process by which inferences, themes, etc., were identified and developed, including researchers involved in data analysis; usually references a specific paradigm or approach; rationale^a^ | 7 |
| S15 Techniques to enhance trustworthiness | Techniques to enhance trustworthiness and credibility of data analysis (e.g., member checking, audit trail, triangulation); rationale^a^ | 7 |
| **Results/Findings** |  |  |
| S16 Synthesis and interpretation | Main findings (e.g., interpretations, inferences, and themes); might include development of a theory or model, or integration with prior research or theory | 7-13 |
| S17 Links to empirical data | Evidence (e.g., quotes, field notes, text excerpts, photographs) to substantiate analytic findings | 8-13 |
| **Discussion** |  |  |
| S18 Integration with prior work, implications, transferability, and contribution(s) to the field | Short summary of main findings; explanation of how findings and conclusions connect to, support, elaborate on, or challenge conclusions of earlier scholarship; discussion of scope of application/generalizability; identification of unique contribution(s) to scholarship in a discipline or field | 14-16 |
| S19 Limitations | Trustworthiness and limitations of findings | 16-17 |
| **Other** |  |  |
| S20 Conflicts of interest | Potential sources of influence or perceived influence on study conduct and conclusions; how these were managed | 2 |
| S21 Funding | Sources of funding and other support; role of funders in data collection, interpretation, and reporting | 2 |

^a^The rationale should briefly discuss the justification for choosing that theory, approach, method, or technique rather than other options available, the assumptions and limitations implicit in those choices, and how those choices influence study conclusions and transferability. As appropriate, the rationale for several items might be discussed together.

**Online Resource 2. Interview Protocol**

**Introductory questions**

1. Describe what an average working shift might look like for you – i.e., what you are doing from when you clock in to when you clock out.
2. Consider the lifecycle of a typical trauma case – from when a patient has just entered the trauma bay, to the point where they are discharged. Could you describe the various points of this cycle where you would be interacting with EHR records for this patient (both in terms of writing up records as well as reviewing and analyzing their records)?
3. During an average shift, how much time approximately would you spend interacting with [EHR system] EHR?
   - How much time do you approximately spend *inputting* data into [EHR system]?
   - How much time do you approximately spend *reviewing and analyzing* data from [EHR system]?

**Generating and inputting EHR data**

4. During a typical work shift, when would you be typing up notes and inputting them into [EHR system]?
   - Typically, where are you located when you are typing up your notes?
   - How long would you take on average to type up a single note?
5. Could you think of a recent case where you felt you did a particularly good job of writing notes?
   - What was it about this case that made it possible to take notes effectively?
   - What are some barriers that prevent you from taking notes in this way all the time?
6. To what extent is effective and high-quality note-taking an important aspect of what it means to be a good [surgeon, resident, nurse]?
   - Do you consider yourself to be, in general, effective in your documentation practices? Why/why not?
   - Do you think your note-taking practices could, at some point in the future, be entirely automated? Why/why not?
7. In what ways could your current documentation practices be streamlined/improved?
   - Can you think of any particular improvements to the current [EHR system] that could significantly improve your note-taking workflows?

**Analyzing and using EHR data**

8. During a typical work shift, when and where would you be reading/analyzing/reviewing EHR data?
   - What specifically are you looking for when you review EHR data?
9. Can you think of a recent case where you felt like you were able to get exactly what you were looking for when reviewing records for a particular case?
   - What was it about this case that made your interactions with EHR so effective?
   - What are some barriers that prevent you from analyzing EHR data in this way all the time?
10. To what extent is the ability to interpret and analyze EHR data an important aspect of what it means to be a good [surgeon/resident/nurse]?
    - Do you consider yourself, in general, to be good at analyzing EHR data? Why/why not?
11. Suppose we were to implement a new dashboard on [EHR system] to provide AI-based risk predictions of certain key surgical rescue outcomes at a glance.
    - Of the input variables that are already collected and presented on [EHR system], which variables are most important to you in interpreting EHR data and predicting surgical rescue outcomes?
    - What kinds of risk predictions would you find most useful to be included in this dashboard?
    - Do you think such a dashboard would significantly improve your workflow? Why/Why not?
    - How would you evaluate if these AI-generated risk predictions could be incorporated into your clinical decision-making?

**General Perceptions of [EHR system] and its Impact on Work**

12. Do you think that the time you spend each day interacting with [EHR system] is appropriate/reasonable?
    - Do you wish you could spend more/less time interacting with [EHR system]?
13. What are some of your biggest frustrations when interacting with [EHR system]?
14. Are you generally satisfied with EHR workflows at [the institution]? Why/why not?
15. Have you used any clinical decision support tools that provide predictions or recommendations based on EHR data?
    - [If yes] What did you find useful about these tools?
    - [If yes] What did you find lacking in these tools?

**Closing Questions**

16. Are there any important issues related to EHR documentation and its impact on your work that you feel we have missed out in the questions we are asking?
17. Is there anyone else that you think we should talk to as part of this study?

**Demographics**

18. How long have you been working as a [trauma surgeon/nurse/resident]?
    - How long have you worked in each of the three ACS sections: trauma, EGS, SICU?
19. How long have you been at [the institution]?
20. Did you use [EHR system] prior to [the institution]? How long have you been using [EHR system] for?
